# C-Type Lectin in *Chlamys farreri* (CfLec-1) Mediating Immune Recognition and Opsonization

**DOI:** 10.1371/journal.pone.0017089

**Published:** 2011-02-15

**Authors:** Jialong Yang, Lingling Wang, Huan Zhang, Limei Qiu, Hao Wang, Linsheng Song

**Affiliations:** 1 The Key Laboratory of Experimental Marine Biology, Institute of Oceanology, Chinese Academy of Sciences, Qingdao, China; 2 Graduate School, Chinese Academy of Sciences, Beijing, China; University of Pittsburgh, United States of America

## Abstract

**Background:**

C-type lectins are a superfamily of Ca^2+^ dependent carbohydrate-recognition proteins that play significant diverse roles in nonself-recognition and clearance of invaders. Though they are well characterized in vertebrates, the study of the potential function and mechanism of C-type lectins in invertebrate immunity is still in its infancy.

**Methodology:**

A C-type lectin (CfLec-1) from scallop *Chlamys farreri*, a dominant cultured mollusk species in China, was selected to investigate its mRNA expression, localization and the possible functions in innate immunity in the present study. After scallop was stimulated by three typical PAMPs, the mRNA expression of CfLec-1 in hemocytes was poles apart. It was significantly up-regulated (p<0.01) after scallops were stimulated by LPS or β-glucan, but significantly down-regulated (p<0.01) after PGN stimulation. The binding ability of recombinant CfLec-1 (designated as rCfLec-1) towards eight PAMPs was investigated subsequently by PAMPs microarray, which revealed rCfLec-1 could bind LPS, PGN and mannan *in vitro*, indicating CfLec-1 served as a PRR involved in the pathogen recognition. Immunofluorescence assay with polyclonal antibody specific for CfLec-1 revealed that CfLec-1 was mainly located in the mantle and gill of the scallop. CfLec-1 could bind to the surface of scallop hemocytes and recruited hemocytes to enhance their encapsulation *in vitro*, and this process could be specifically blocked by anti-rCfLec-1 antibody. Meanwhile, rCfLec-1 could also enhance the phagocytic activity of scallop hemocytes against *Escherichia coli*.

**Conclusions:**

The results clearly suggested that CfLec-1 in *C. farreri* not only served as a PRR involved in the PAMPs recognition, but also functioned as an opsonin participating in the clearance of invaders. It is therefore suspected that CfLec-1 could be an attachment-molecule to nonself-agents acting as an alternative to immunoglobulin in vertebrates.

## Introduction

Innate immune response is a common feature of metazoan organisms, which protect hosts against pathogens and opportunistic microbial infections. Invertebrates are devoid of an adaptive immune system and they exclusively rely on innate immune reactions for theirs defense [1], [2]. The first and crucial step of innate immune response is to recognize highly conserved structures present in a large group of microorganisms but absent in responding species, referring to as pathogen-associated molecular pattern (PAMPs). And the receptors in innate immune system, which have evolved to recognize PAMPs, are known as pattern recognition receptors (PRRs) [3], [4]. At least six groups of PRRs, including peptidoglycan recognition protein (PGRP), Toll-like receptor (TLR), thioester-containing protein (TEP), lipopolysaccharide and β-1, 3-glucan binding protein (LGBP), C-type lectin (CTL) and galectin (GALE), have been identified from invertebrates [5]. Among of them C-type lectin is a well known PRR which can recognize and bind to terminal sugars on the microorganisms and takes part in the immune recognition against pathogens [5], [6].

C-type lectins are a superfamily of Ca^2+^ dependent carbohydrate-recognition proteins, which play significant roles in non-self recognition and clearance of invaders, either as cell surface receptors for microbial carbohydrates or as soluble proteins existing in tissue fluids [5], [6]. They involve in several innate immune responses [7], such as proPO activation [8], [9], nodule formation [10], phagocytosis [11] and antibacterial activity [12]. All the carbohydrate-recognition behaviors of C-type lectins are benefit from their carbohydrate-recognition domain (CRD) of ∼130 amino acid residues [13], [14]. In each CRD, there is a characteristic double-loop stabilized by two highly conserved disulfide bridges located at the bases of the loops, as well as a set of conserved hydrophobic and polar interactions. There are four Ca^2+^-binding sites identified from CRDs of different species, among which the Ca^2+^-binding site 2 is structurally conserved and involved in carbohydrate-binding activity [15].

In recent years, more and more C-type lectins have been identified from invertebrates, especially from insects and crustaceans [8], [10], [16]–[19]. Compared with those in arthropods, research on mollusk C-type lectins is in its infancy, though several C-type lectins have been identified from mollusc and most of them are believed to involve in immune defence [20], [21]. In our previous study, several C-type lectins genes (CfLec-1 - CfLec-4) were identified from Zhikong Scallop *Chlamys farreri*, an important economic bivalve specie cultured widely in the northern coast of China, and they were proved to involve in the immune response against *Vibrio anguillarum* [22]–[25], but the possible functions they performed and the underlined mechanism were still not well understood. In the present study, CfLec-1 was selected for further study in order to (1) examine its PAMPs recognition and binding pattern, (2) detect its localization in the tissues of *C. farreri*, (3) characterize the opsonization mediated by CfLec-1, including encapsulation and phagocytosis, and (4) get further understanding of the immune system of *C. farreri*.

## Materials and Methods

### Ethics statement

The scallops used in the present study are marine cultured animals, and all the experiments are conducted according to the regulations of local and central government. Female Wistar rats were from Qingdao institute for the control of drug products (Qingdao, China), and the animal experiments were approved by the Animal Care and Use Committee at Qingdao institute for the control of drug products with a permit number of SCXK (Shandong) 20090007, which complied with the National Institute of Health Guide for the Care and Use of Laboratory Animals.

### Immune stimulation of scallop

Adults of scallop *C. farreri* with an average 55 mm of shell length were collected from a farm in Qingdao, Shandong Province, China, and maintained in the aerated seawater at 15°C for a week before processing.

Two hundred scallops were employed for the PAMPs stimulation experiment. The scallops were randomly divided into 5 groups and each group contained 40 individuals. Four groups receiving an injection of 50 µL phosphate buffered saline (PBS, 0.14 M sodium chloride, 3 mM potassium chloride, 8 mM disodium hydrogenphosphate dodecahydrate, 1.5 mM potassium phosphate monobasic, pH 7.4), LPS from *Escherichia coli* 0111:B4 (Sigma-Aldrich, 0.5 mg ml^−1^ in PBS), PGN from *Staphylococcus aureus* (Sigma-Aldrich, 0.8 mg ml^−1^ in PBS), and β-glucan from *Saccharomyces cerevisiae* (Sigma-Aldrich, 1.0 mg ml^−1^ in PBS), respectively. The untreated group was employed as blank group. After treatment, the scallops were returned to water tanks and 5 individuals were randomly sampled at 3, 6, 12, 24 and 48 h post-injection. The hemolymphs were collected, and centrifuged at 500 ×g, 4°C for 10 min to harvest the hemocytes for RNA preparation.

### Expression patterns of CfLec-1 post PAMPs stimulation

Total RNA was extracted using Trizol reagent according to the manufacture's protocol (Invitrogen). The first-strand synthesis was carried out based on Promega M-MLV RT Usage information using the DNase I (Promega)-treated total RNA as template and oligo (dT)-adaptor as primer (Table 1). The reaction mixtures were performed at 42°C for 1 h, terminated by heating at 95°C for 5 min. cDNA mix was diluted to 1∶100 and stored at −80°C for subsequent SYBR Green fluorescent quantitative real-time PCR (RT-PCR).

**Table 1:** Primers used in the present study.

Primer	Sequence (5′—3′)
Oligo(dT)-adaptor	GGCCACGCGTCGACTAGTACT_17_
*CfLec-1* RTF (forward)	CAACCTGTTCTATATCTGCGAG
*CfLec-1* RTR (reverse)	GATCTGTTGGCTGATTTCAC
*β-actin* AF (forward)	CAAACAGCAGCCTCCTCGT
*β-actin* AR (reverse)	CTGGGCACCTGAACCTTTCGTT

One pair of gene-specific primer for CfLec-1 (Table 1) was used to amplify product of 228 bp from cDNA. The PCR product was sequenced to verify the specificity of RT-PCR. Two β-actin primers, AF and AR (Table 1), were used to amplify a 94-bp fragment as an internal control to verify the successful transcription and to calibrate the cDNA template for corresponding scallop samples.

Real-time PCR amplification was carried out in an ABI 7300 Realtime Thermal Cycler according to the manual (Applied Biosystems). Dissociation curve analysis of amplification products was performed at the end of each PCR to confirm that only one PCR product was amplified and detected. The 2^−△△^^CT^ method was used to analyze the expression level of CfLec-1 [26]. All data were given in terms of relative mRNA expressed as mean ± SE (N = 4). The data were subjected to one-way analysis of variance (one-way ANOVA) followed by an unpaired, two-tailed t-test. Differences were considered significant at P<0.05 and extremely significant at P<0.01.

### Preparation of recombinant protein of CfLec-1

The strains *E. coli* BL21(DE3)-pLysS with recombinant plasmid (pET-32a-CfLec-1) [22] and the negative control pET-32a (Novagen) vector without insert fragment which could express a thioredoxin (Trx with 6×His-tag in the prokaryotic express system), were incubated in LB medium (containing 50 mg ml^−1^ ampicillin) at 37°C with shaking at 220 rpm. When the culture mediums reached OD600 of 0.5–0.7, the cells were incubated for 4 additional hours with the induction of IPTG at the final concentration of 1 mmol L^−1^. The recombinant protein CfLec-1 and Trx (designated rCfLec-1 and rTrx) were purified by a Ni^2+^ chelating Sepharose column, pooled by elution with 400 mmol L^−1^ imidazole under denatured condition (8 mol L^−1^ urea). The purified protein was refolded in gradient urea-TBS glycerol buffer (50 mmol L^−1^ Tris–HCl, 50 mmol L^−1^ NaCl, 10% glycerol, 2 mmol L^−1^ reduced glutathione, 0.2 mmol L^−1^ oxide glutathione, a gradient urea concentration of 6, 5, 4, 3, 2, 1, 0 mol L^−1^ urea, pH 8.0; each gradient at 4°C for 12 h). The resultant proteins were separated by reducing 15% SDS-polyacrylamide gel electrophoresis (SDS-PAGE), and visualized with Coomassie bright blue R250. The concentration of purified rCfLec-1 and rTrx was quantified by BCA method [27], respectively. The obtained proteins were stored at −80°C before use.

### Preparation of antibodies and Western blotting analysis

For preparation of antibodies, the renatured protein rCfLec-1 was continued to be dialyzed against ddH_2_O before it was freeze concentrated. Trx-tag was removed from the fusion protein by digestion with enterokinase (NEB). rCfLec-1 (without Trx-tag) was immuned to 6 weeks old rats to acquire polyclonal antibody as the method discribed by Cheng [28]. The antiserum was purified by the Ampure PA kit (Amersham) following the manufacturer's protocol, and then the purified Ig was labeled with Cy3 (GE Healthcare) according to the suggestion of the manufacturers and used as microarray detection antibody.

After SDS-PAGE, the samples (4 hours after IPTG added) were electrophoretically transferred onto a 0.45 mm pore nitrocellulose membrane at 200 mA for 5 h. The membrane was blocked with PBS containing 3% BSA at 37°C for 1 h, and incubated with antibodies at 37°C for 1 h, then washed three times with PBS containing 0.05% Tween-20 (PBS-T). Antibody binding was detected with goat-anti-rat Ig-alkaline phosphatase conjugate (Southern Biotech) diluted 1∶4000 in PBS at 37°C for 1 h, and washed three times with PBS-T. Protein bands were stained with freshly prepared substrate solution (100 mM NaCl, 100 mM Tris and 5 mM MgCl_2_, pH 9.5) containing nitroblue tetrazolium (NBT, Sigma) and 5-bromo-4-chloro-3-indolyphosphate (BCIP, Sigma) for 5 min and stopped by washing with distilled water. Rats' pre-immune serum was used as negative control.

### PAMPs binding assay

The PAMPs binding assay of rCfLec-1 was performed by the method of PAMPs microarray according to the previous report with modification [29], [30]. Briefly, a high-precision robot designed to produce cDNA microarrays (GMS 417 Arrayer; Genetic Microsystems, Inc., Woburn, MA) was utilized to spot PAMPs antigens onto the glass slides precoated with nitrocellulose polymer (FAST Slides; Schleicher & Schuell, Keene, NH). PAMPs were dissolved in PBS in concentrations as specified in Table 2, and printed with spot sizes of ∼150 µm and center to center intervals of 150 µm. The printed PAMPs microarrays were air-dried and stored at 4°C without desiccant before application. Immediately before use, the printed PAMPs microarrays were rinsed with PBS-T and then blocked by incubating the slides in 3% BSA in PBS at 37°C for 1 h. After three times' washing with PBS-T, 20 µl of rCfLec-1 in the concentration of 100 µg mL^−1^ was added onto the microarrays, and the same dose of rTrx was set as a control. After incubated at 18°C for 2 h, the microarrays were washed three times with PBS-T, and then 20 µl of Cy3-conjugated anti-CfLec-1 antibody (1∶300 diluted in PBS) was added and incubated at 37°C for 1 h. After three times' rinses, the images of the microarrays were obtained using a laser chipscanner (Capitalbio Corporation, China) at 532 nm, and analyzed with EcoscanCHS software to quantify florescence intensity.

**Table 2:** Characteristic of the samples print on the microarrays.

Number	Sample name	Source	Concentration	Purpose
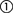	lipopolysaccharides	*E. coli* (Sigma)	1 mg mL^−1^	PAMP
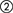	peptidoglycan	*S. aureus* (Sigma)	1 mg mL^−1^	PAMP
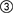	yeast glucan	*S. cerevisiae* (Sigma)	1 mg mL^−1^	PAMP
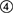	β-1,3-glucan	*Euglena gracilis* (Sigma)	1 mg mL^−1^	PAMP
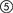	mannan	*S. cerevisiae* (Sigma)	1 mg mL^−1^	PAMP
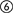	lipoteichoic acids	*S. aureus* (Sigma)	1 mg mL^−1^	PAMP
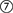	CpG ODN	Digested from our constructed plasmid	20 µg mL^−1^	PAMP
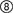	poly I:C	Sigma	1 mg mL^−1^	PAMP
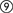	rabbit anti-rat IgG	rabbit	0.1 mg mL^−1^	Positive control
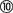	PBS-glycerol	-	40% (v/v)	Negative control

The dose-dependent PAMPs binding activity of rCfLec-1 was measured according to previous report with modification [31]. Briefly, 20 µg of PAMPs were used to coat 96-well microtiter plate (Costar) in 100 µl of carbonate-bicarbonate buffer (50 mM, pH 9.6) and incubated overnight at 4°C. The wells were washed three times with PBS-T and then blocked with 200 µl of 3% BSA in PBS at 37°C for 1 h. The plate was washed three times and 100 µl several concentrations of rCfLec-1 was then added to the wells in the presence of 5 mM CaCl_2_ and 0.1 mg ml^−1^ BSA. The same concentration of rTrx was used as a control. After incubated at 18°C for 2 h, the plate was washed three times with PBS-T, and then 100 µl of rats' immune serum of rCfLec-1 (diluted 1∶1000 in PBS) was added to the wells as the first antibody. After incubation at 37°C for 1 h, the plate was washed again and 100 µl of goat-anti-rat Ig-alkaline phosphatase conjugate (Southern Biotech) diluted 1∶4000 in PBS was added as second antibody and incubated at 37°C for 1 h. After the last washing, 100 µl of 0.1% (w/v) p-nitrophenyl phosphate (pNPP, Sigma) in 50 mM carbonate-bicarbonate buffer (pH 9.8) containing 0.5 mM MgCl_2_ was added to each well and incubated at room temperature in dark for 30 min. The reaction was stopped by adding 50 ml of 2 M NaOH per well and the absorbance was measured with an automatic ELISA reader at 405 nm (Molecular Devices). The wells filled with 100 µl of TB were used as blank. Non-immune rat serum instead of immune serum as first antibody was set as negative control. Each experiment was repeated in triplicate. Samples with P (sample) – B (blank)/N (negative) – B (blank) >2.1 were considered positive.

### Immunofluorescence detection of CfLec-1 in tissues

The hemolymph from five scallops (about 0.5 ml per individual) collected by a syringe containing pre-cooled (4°C) modified Alsever solution (0.12 M glucose, 0.03 M sodium citrate, 9 mM EDTA and 0.38 M NaCl, pH 7.2) as an anticoagulant were pooled together and immediately centrifuged at 500 ×g, 4°C for 10 min to harvest the hemocytes. The hemocyte pellet was resuspended in PBS, and deposited on clean slides (a drop on each) in the wet chamber for 1 h. After hemocytes settled in monolayers on slides, the PBS was sipped up using a filter paper, followed by fixing the slides in acetone for 15 min before being stored at −20°C until use.

The gill, adductor muscle, gonad, hepatopancreas, mantle and kidney of scallops dissected according to Lin [32] were embedded in tissue freezing medium (Leica) and stored at −20°C. Continuous 7 µm thick frozen sections of each sample were cut using a Leica CM1900 cryostat at −20°C and transferred onto glass slides, which were pre-coated with gelatin solution (Gelatin 5 g L^−1^, CrK(SO_4_)_2_ ·12H_2_O 0.5 g L^−1^) and fixed with pre-cooled acetone for 15 min. These tissues cryosections were stored at −20°C until use.

The slides of hemocytes and tissues were covered with 20 µl antibody of rCfLec-1 (diluted 1∶1000 in PBS) as primary antibody and incubated at 37°C for 1 h in a moisture chamber. After three times washing with PBS-T, the slides were incubated at 37°C for 1 h with fluorescein isothiocyanate conjugated goat anti-rat immunoglobulins serum diluted at 1∶200 with PBS (Southern Biotech), contained 1 µg ml^−1^ Evan's blue dye (EBD, Fluka) as the counterstain. Finally, the slides were washed three times and mounted in buffered glycerin for observation by fluorescence microscope. Rats' preimmune serum was used as negative control.

### *In vitro* encapsulation

*In vitro* encapsulation assay was performed according to previous report [33]. Briefly, Ni-NTA agarose beads (Qiagen) were equilibrated in TBS containing 5 mM CaCl_2_, and then renatured His-tagged rCfLec-1 and rTrx (as a control protein) were added and incubated with agarose beads in a 1.5 ml tube with shaking at 4°C overnight, respectively. Protein-coated beads were washed with TBS four times, each for 5 min, and suspended in TBS at 100–120 beads per microliter. A 48-well cell culture plate (Costar) was treated with 1% agarose (Qiagen). The hemolymph was withdrawn by a sterile syringe from the adductor muscle and simultaneously diluted (1∶3) in anticoagulant. Hemocytes were suspended in 200 µl of Leibovitz L-15 medium (Sigma). The diluted hemolymph was added to each well of the agarose-coated plate. Hemocytes were allowed to settle down for at least 10 min, then 1 µl (100–120 beads) of the protein-coated agarose beads was added to each well, and the plate was incubated at 18°C. Encapsulation of the agarose beads was observed after 6 and 24 h incubation, respectively. For each recombinant protein, assay was performed in three different wells for statistic analysis.

To test whether *in vitro* encapsulation was specifically incited by C-type lectin, 5 µl protein-coated beads were placed in a microcentrifuge tube containing 50 µl of rat anti- rCfLec-1 antiserum in a total of 100 µl TBS (pH 7.5), and the mixture was incubated at 4°C overnight with shaking. Then the beads were washed with TBS and suspended in 5 µl TBS. *In vitro* encapsulation assay was performed as described above.

### Phagocytosis assay

Phagocytosis assay was performed according to previous method with modification [34]. Briefly, hemolymph was collected from five scallops with a syringe, and mixed immediately with equal volume of pre-chilled anticoagulant (Tris-HCl 50 mM; glucose 2%, NaCl 2%; EDTA 20 mM; pH 7.4), and then centrifuged at 800 g for 10 min. The hemocytes were resuspended with 200 µl TBS buffer (Tris-HCl 50 mM; CaCl_2_ 5 mM), 100 µg mL^−1^ rTrx in TBS buffer and 100 µg mL^−1^ rCfLec-1 in TBS buffer, respectively. The hemocytes were incubated at 18°C for 30 min, then 5 µl *E. coli* (OD_600_ = 0.4, suspended in Tris-HCl) was added into each hemocytes suspension, and incubated at 18°C for another 1 h. About 50 µl of the mixture was mounted onto a glass slide and put into a moist chamber for 1 h. The slides were washed with Tris-HCl, air-dried, fixed with methyl alcohol and stained with Giemsa. The phagocytic activity of hemocyte attached to the slide was measured using a light microscope. Two hundred hemocytes on each slide were counted. Phagocytic rate (PR) and phagocytic index (PI) representing the phagocytic activities were expressed as following: PR =  (phagocytic hemocytes)/(total hemocytes)×100%; PI = average number of bacteria in phagocytic hemocyte. For each treatment, assay was performed in three different slides for statistic analysis.

## Results

### The mRNA expression patterns post PAMPs stimulation

**Figure 1:**
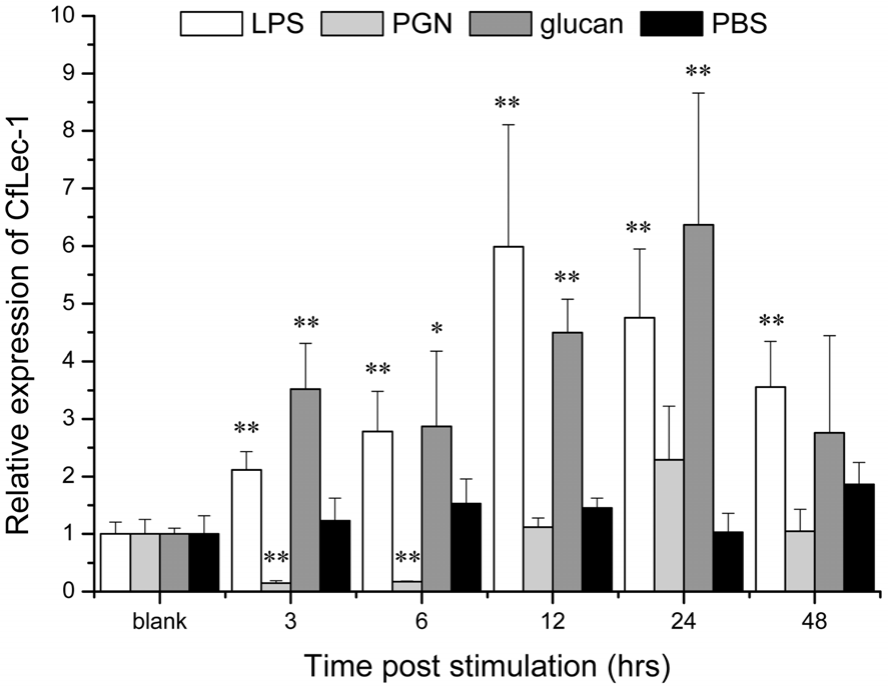
Temporal expression of CfLec-1 mRNA relative to β-actin was analyzed by realtime PCR in scallop hemocytes after LPS, PGN, β-glucan and PBS challenge for 3, 6, 12, 24 and 48 h. The values are shown as mean ± SE (N = 4). (*: P<0.05, **: P<0.01).

Real-time PCR was used to monitor the mRNA expression of CfLec-1 transcripts in adult animals stimulated by three typical PAMPs (Fig. 1). In the LPS stimulated group, the mRNA expression of CfLec-1 was significantly (P<0.01) up-regulated, and it reached the maximal level (5.9-fold compared with blank group) at 12 h after challenge. Similar significant (P<0.01) up-regulation expression of CfLec-1 transcript was also observed in the β-glucan stimulated group, though the maximal level, which was 6.4-fold compared with blank group, was observed at 24 h after β-glucan stimulation. On the contrary, the mRNA expression in the PGN stimulated group was significantly (P<0.01) down-regulated, and it exhibited a level of 0.14 and 0.17-fold compared with blank group at 3 h and 6 h after stimulation, respectively, then the expression increased to the original level and last to 48 h after challenge. In the control group, there was no significant change of CfLec-1 expression during the whole experiment after PBS injection.

### Preparation of recombinant protein and western blotting analysis

**Figure 2:**
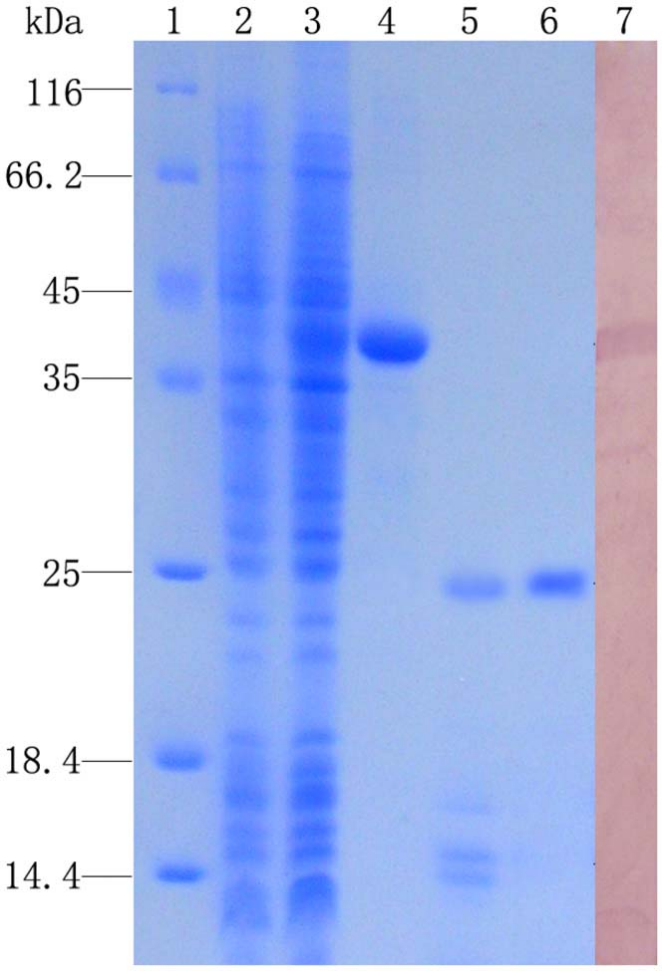
SDS-PAGE and Western-blot analysis of rCfLec-1. Lane 1: protein molecular standard; lane 2: negative control for rCfLec-1 (without induction); lane 3: induced rCfLec-1; lane 4: purified rCfLec-1; lane 5: production of rCfLec-1 digested by enterokinase; lane 6: rCfLec-1 with out Trx tag; lane 7: western-blot of rCfLec-1.

After IPTG induction, the whole cell lysate of *E. coli* BL21 (DE3)-pLysS with pET-32a-CfLec-1 was analyzed by SDS-PAGE. A distinct band with molecular weight of ∼40 kDa was revealed, which was consistent with the predicted molecular mass (Fig. 2, line 3 and 4). Meanwhile, the transformant with parent vector expressed a unique 20 kDa product representing rTrx (data not shown).

To develop antibody, fusion protein rCfLec-1 was digested with enterokinase to remove the Trx-tag. Distinct band with molecular weight of ∼25 kDa was observed in the SDS-PAGE of enterokinase digested mixture, which was consisted with the theoretical molecular weight of CfLec-1. The other bands of 14–17 kDa were the disgested Trx (Fig. 2, line 5). The purified protein (Fig. 2, line 6) was used to prepare antibody.

Western blotting was carried out to identify the specificity of antibody. A clear reaction band of CfLec-1 with high specificity was revealed (Fig. 2), and few band of non-specific was visible (Fig. 2, line 7). As negative control, no visible reaction band was detected in group of rats' pre-immune serum (data not shown).

### PAMPs binding assay

**Figure 3:**
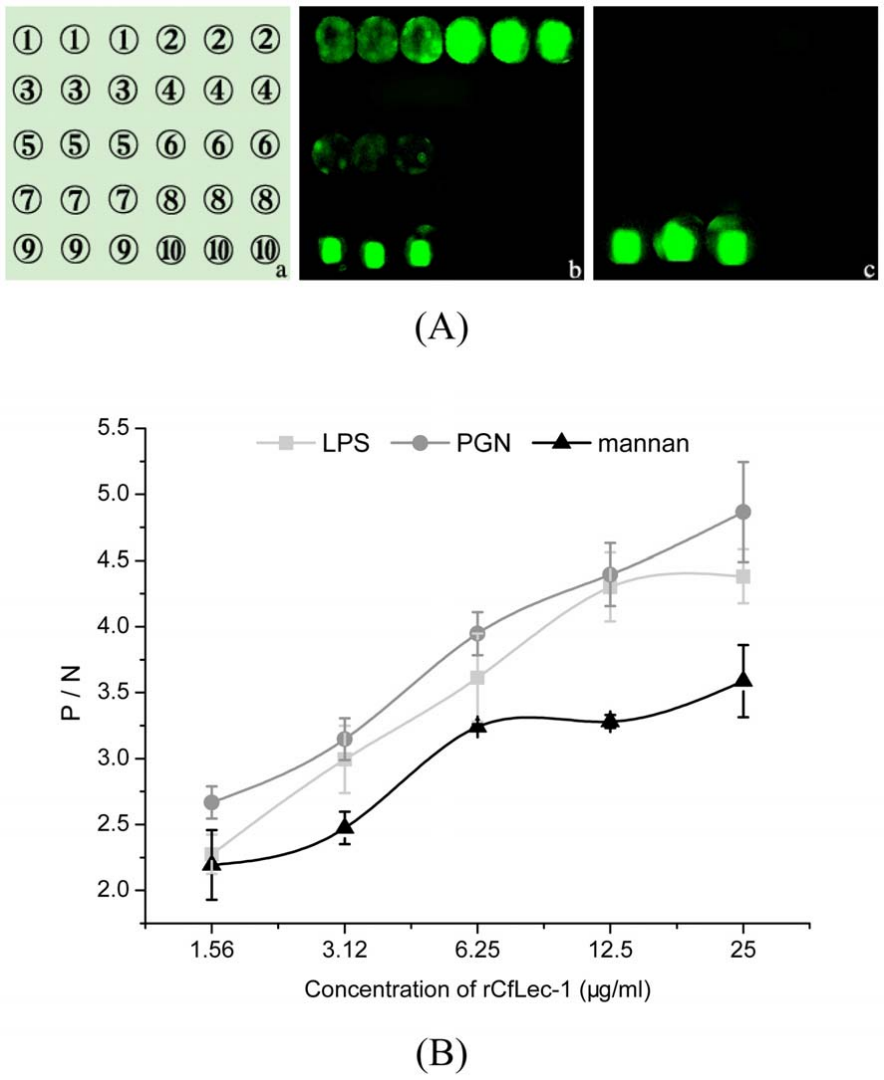
PAMPs binding assays. (A) PAMPs binding assay with microarrays. Samples were print onto the glass slides as the order showed in Table 2. The PAMPs microarrays were blocked by BSA, then rCfLec-1was added, and rTrx was set as a control. The interaction was detected by Cy3-conjugated anti-CfLec-1 antibody. (B) The dose-dependent of PAMPs binding by the method of ELISA. Plates were coated with 20 µg of PAMPs, respectively, which were washed and incubated with several concentrations of rCfLec-1 and rTrx in the presence of 5 mM CaCl_2_ at 18°C for 3 h and detected with rat polyclonal antiserum and goat-anti-rat Ig-alkaline phosphatase conjugate. Samples with P/N>2.1 were considered positive. Results are representative of average three such experiments.

PAMPs binding assay was performed by the method of PAMPs microarrays, and the binding activity was calculated by the signal value of fluorescence. The rCfLec-1 could bind PGN and LPS obviously with intense signal value of fluorescence, bind mannan weakly with faint signal value of fluorescence. It could not bind other PAMPs including yeast glucan, β-1,3-glucan, LTA, CpG ODN and poly I:C (Fig. 3, A, b). As a protein control, rTrx could not bind any PAMPs used in this experiment (Fig. 3, A, c).

The dose-dependent PAMPs binding activity of rCfLec-1 was examined by the method of ELISA. The binding activity was recorded as P/N at 405 nm, and the samples with P/N >2.1 were considered as positive. All the value of P/N were higher then 2.1 at a lower concentration of rCfLec-1 (1.56 µg ml^−1^) in each PAMP group, and the values were increased corresponding with the increasing of rCfLec-1 concentration (Fig. 3, B), which suggesting the binding activities of rCfLec-1 towards PGN, LPS and mannan exhibited dose-dependent effect. As a protein control of C-type lectin, rTrx could not bind any PAMPs used in this experiment (data not shown).

### Immunofluorescence detection of CfLec-1 in tissues

**Figure 4:**
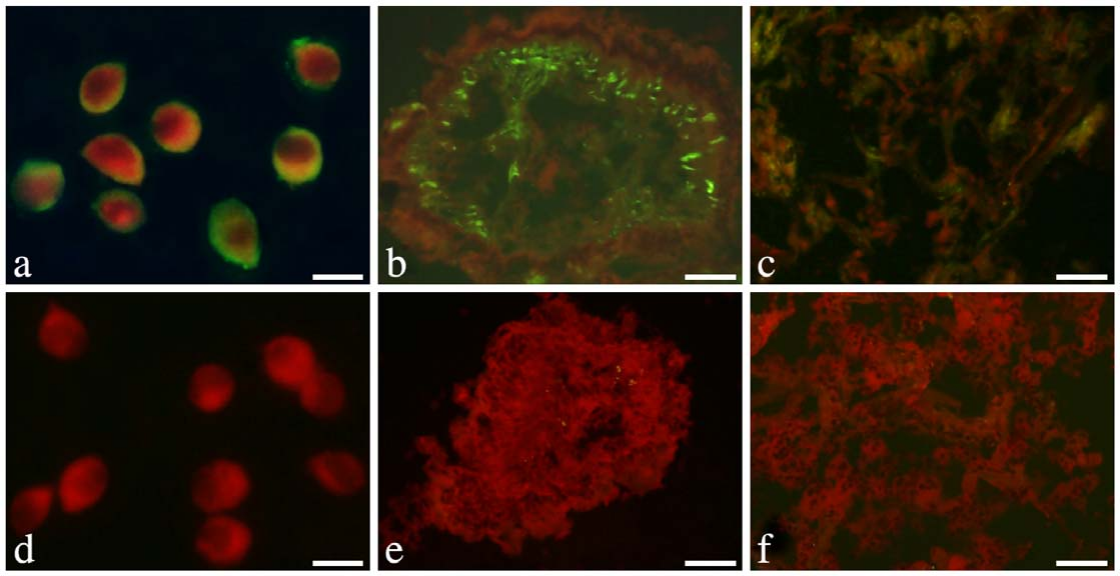
Localization of endogenous CfLec-1 in different tissues. Hemocytes or 7 µm thick frozen sections of gill, adductor muscle, gonad, hepatopancreas, mantle and kidney were fixed with acetone and then incubated with rat polyclonal antiserum to CfLec-1. Binding of antibody to CfLec-1 was visualized by FITC-labeled secondary antibody (green), and the whole tissues were stained with EBD (red). a and d: hemocytes, bar = 10 µm; b and e: mantle, bar = 50 µm; c and f: gill, bar = 50 µm.

Localization of endogenous CfLec-1 in different tissues was detected by the method of immunofluorescence. CfLec-1 was observed on the surface of scallop hemocytes (Fig. 4, a), though it was predicted to be secreted into hemolymph. Besides, CfLec-1 was also detected to be located in the mantle (Fig. 4, b) and gill (Fig. 4, c). No fluorescence staining was observed in other tissues detected, including kidney, gonad, hepatopancreas and muscle (data not shown). There was no fluorescence staining was observed in negative control groups (Fig. 4, d, e and f).

### *In vitro* encapsulation

**Figure 5:**
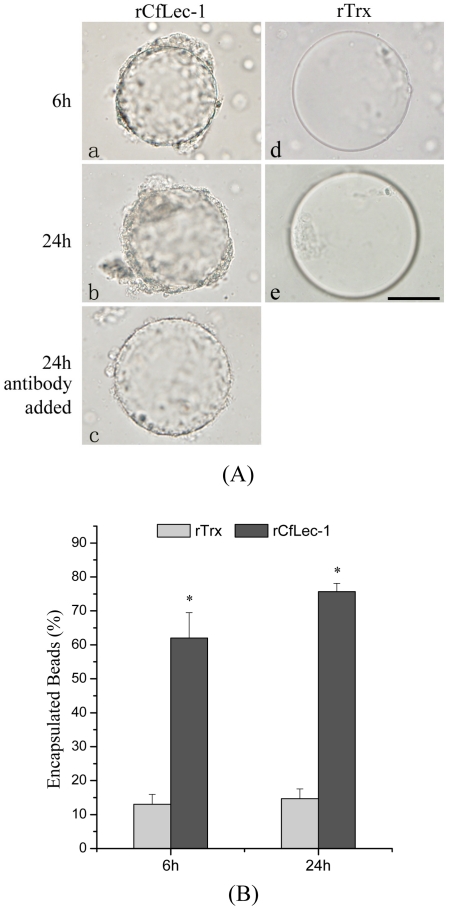
rCfLec-1 promotes encapsulation by scallop hemocytes. Nickel agarose beads coated with rCfLec-1, or rTrx were incubated with scallop hemocytes. Encapsulation of the protein-coated beads was observed by microscopy after 6 and 24 h incubation. rCfLec-1-coated agarose beads were pre-incubated with antibody of rCfLec-1, and encapsulation of the antibody-bound beads was performed as above and observed. (A) Protein-coated beads were encapsulated by scallop hemocytes, and hemocyte encapsulation of the lectin-coated beads was block by antiserum specific for rCfLec-1, bar = 50 µm. (B) The numbers of encapsulated at 6 h and 24 h beads. The columns represent the mean of three individual counts ± S. E. M.

To investigate whether rCfLec-1 could initiate encapsulation, an *in vitro* encapsulation assay using protein-coated agarose beads was performed. Most of the beads (62%) coated with rCfLec-1 were encapsulated by scallops' hemocytes within 6 h of incubation (Fig. 5, A, a; B), while few beads (13%) coated with rTrx were encapsulated (Fig. 5, A, d; B). After 24 h of incubation, the rates of encapsulated beads coated with rCfLec-1 increased to 75.6%, while only 14.7% of the beads coated with rTrx were encapsulated (Fig. 5, B).

To test whether enhanced encapsulation by rCfLec-1 was caused by direct interaction between rCfLec-1 and hemocyte surface molecules, lectin-coated beads were pre-incubated with rat polyclonal antibody to block the surface. After pre-incubation of antibody with the lectin-coated beads, the adhesion of hemocyte was blocked effectively (Fig. 5, A, c).

### Phagocytosis assay

**Figure 6:**
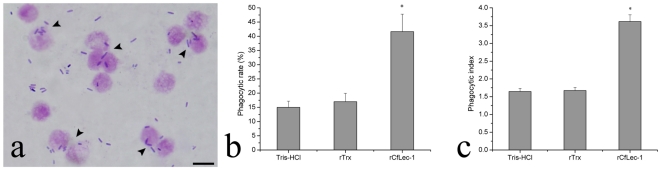
Phagocytosis enhanced by rCfLec-1. The hemocytes were resuspended with TBS buffer, rTrx in TBS buffer and rCfLec-1 in TBS buffer, respectively. The hemocytes were incubated for 30 min, then *E. coli* was added into each hemocytes suspension for another 1 h. The mixture was mounted onto a glass slide and stained with Giemsa. The phagocytic activity of hemocyte was measured using a light microscope. Two hundred hemocytes on each slide were counted. Phagocytic rate (PR)  =  (phagocytic hemocytes)/(total hemocytes)×100%; phagocytic index (PI)  = average number of bacteria in phagocytic hemocytes. For each treatment, assay was performed in three different slides for statistic analysis.

CfLec-1 could significantly enhance the phagocytic activity of scallop hemocytes (Fig. 6). The phagocytized *E. coli* was clearly observed in the cytoplasm of the hemocytes (Fig. 6, a). Compared with Tris-HCl and rTrx, rCfLec-1 significantly enhanced (P<0.05) the phagocytic activity against *E. coli*, both in phagocytic rate and phagocytic index. The phagocytic rate in rCfLec-1 group was 41.7%, while that in Tris-HCl and rTrx group were 15% and 16.7%, respectively (Fig. 6, b). And the phagocytic index was also increased from 1.64 in Tris-HCl group to 3.62 in rCfLec-1 group (Fig. 6, c).

## Discussion

C-type lectins play significant roles in both adaptive immunity and innate immunity to defense against pathogen infection [7], [35], [36] by involving in various immune responses, such as pathogen recognition [37]–[39], cellular adhesion [40]–[43], antigen uptake [44], [45] and complement pathway activation [38], [46], [47]. However, to our knowledge, the possible immune functions and the underlined mechanism of mollusk C-type lectin are still not well understood. In the present study, one of the C-type lectins from *C. farreri*, CfLec-1, was selected to investigate its functions in PAMPs recognition and opsonization. The results clearly indicated that CfLec-1 not only served as a PRR involving in the PAMPs recognition, but also functioned as an opsonin participating in the clearance of invaders.

Immune recognition, which discriminates self from the potentially harmful non-self, is the premier and crucial step in the innate immunity. The predominant PAMPs recognition and binding ability of C-type lectin make it an indispensable molecule in the first defense line against pathogen infection [22], [25]. In invertebrates,several C-type lectins have heen characterized and their mRNA expression could be induced by the stimulation of PAMPs [17], [48], [49]. In the present study, the mRNA expression of CfLec-1 was significantly up-regulated after scallops were stimulated by LPS or β-glucan, indicating CfLec-1 was involved in the immune response induced by these two PAMPs. PAMPs binding assay revealed that rCfLec-1 could bind LPS from Gram-negative bacteia and PGN from Gram-positive bacteria, indicating that CfLec-1 could recognize PAMPs and served as PRR. Whereas, rCfLec-1 could not bind β-glucan, though mRNA expression of CfLec-1 was up-regulated significantly after β-glucan injection. The results reminded that CfLec-1 was involved in the recognition of β-glucan, but it could not bind this kind of PAMP independently. Other molecules are probably needed for it to bind β-glucan. Interestingly, rCfLec-1 exhibited strong binding activity towards PGN *in vitro*, but PGN stimulation significantly down-regulated the mRNA expression of CfLec-1. These phenomena tempted us to suspect that the involvement of CfLec-1 in PGN recognition was sophisticated and further research was still needed to better understand the mechanism.

The sugar recognition mediated by C-type lectins benefits from their carbohydrate-recognition domain (CRD) of ∼130 amino acid residues [13], [14]. The CRDs exhibit conservative characteristic in structure, and in each CRD there are a characteristic double-loop stabilized by two highly conserved disulfide bridges, and a Ca^2+^ binding sites 2 which is involved in carbohydrate binding [15]. C-type lectins can bind carbohydrate in a specific manner, which is proved to be determined by the position of hydrogen bond donors and acceptors in Ca^2+^-binding site 2 of CRDs. The CRDs with EPN (Glu-Pro-Asn) motif binds mannose or similar sugar, whereas the QPD (Gln-Pro-Asp) motif prefers galactose or similar sugar [50]. The potential motif in the CRDs of CfLec-1 is EPD, where an Asp (D) substitutes for Asn (N) in the third position. The EPD motif is common in lectins from *C. farreri,* such as the CRD from CfLec-2, CRD2 from the CfLec-3 and the CRD1 from CfLec-4 [22]–[24]. Structurally, with the substitution by Asp, a hydrogen bond donor is replaced with an acceptor. But our present work indicated that this replacement had no effect on activity of PAMPs binding. Similar results were also reported in the cotton bollworm C-type lectin [11]. It was suspected that the PAMPs binding features of invertebrates C-type lectins was more complicated, and other sites in CRDs might also be involved in PAMPs binding.

Different from the broad tissue distribution of other invertebrate C-type lectins, CfLec-1 exhibited a specific distribution profile, and it was located in mantle and gill of the scallop. Mantle is the organ that scallop protrudes out of the shell, and gill takes the responsibility to seize oxygen and alga from the seawater, and the presence of CfLec-1 in these two organs may due to their frequently contact with pathogens. Considering the large number of bacteria in the aquatic environment, this expression of CfLec-1 in susceptible organ suggests the important roles for this molecule in defense of scallops against bacterial infections. In addition, CfLec-1 could also bind to the surface of hemocytes, though it was predicted to be secreted into hemolymph with the instruction of signal peptide. This was similar to some other invertebrates C-type lectins [51], [52], and such binding was believed to be necessary for the opsonization C-type lectin mediated in invertebrates against invaders [53].

Opsonins are host-derived proteins that can increase the efficiency of phagocytosis and diversify the functional repertoire of phagocytes [54], [55]. In vertebrates, two archetypical opsonins – complement fragment C3bi which binds nonspecifically to the surface of foreign particles, and immunoglobulins (Igs) which attach to the target particle by recognizing specific surface epitopes – both have been extensively characterized [54]. Though be devoid of C3bi and Igs in invertebrates, similar opsonization is also reported to be mediate by other molecule, for example PGRP-LC, a peptidoglycan recognition protein is involved in phagocytosis of Gram-negative bacteria in S2 cells [56]. In invertebrates, encapsulation and phagocytosis are frequently observed opsonization against parasite and bacteria, respectively. Several C-type lectins from invertebrates were reported to mediate encapsulation or phagocytosis, such as Immulectins in tobacco hornworm [33] and C-type lectin in cotton bollworm [11]. Phagocytosis is initiated by the binding of bacteria to phagocytes, and the process is facilitated by C3bi or specific Igs in vertebrate. In our present study, CfLec-1 could recognize and bind the bacteria component, as well as bind to the surface of hemocytes, and the multi-directed binding is suspected to be responsible for the phagocytosis mediated by CfLec-1. Encapsulation is another opsonization that is restricted to invertebrates in defense against foreign bodies too large for phagocytosis by individual hemocytes [57], and such immune response requires coordination of both cellular and humoral factors [58]–[60]. Similar to phagocytosis, the coordination of CfLec-1 and hemocytes recruits hemocytes and promote hemocytes encapsulation in our present study. These results indicate that CfLec-1 from *C. farreri* triggers the opsonization of hemocytes against invaders, and it is therefore generally expected that scallop's C-type lectin that is attachment-molecules to non-self-agents act as an alternative to Ig. Since there is no antibody-mediated immunity in mollusk, C-type lectin with diverse specificities may function as non-clonal effectors in the scallop immune system. Taking the PAMPs binding ability of CfLec-1 into consider, we suspected that CfLec-1 involved in immune response against invader with the following mechanism: CfLec-1 firstly bond to the surface of hemocytes, subsequently recognized and bond the invader target on the specific PAMPs, and finally the opsonizations were triggered as a result of the coordination of both cellular and humoral factors.

In our previous studies, 5 C-type lectins have been identified from Zhikong scallop *C. farreri*, and all of them can be induced by *Listonella anguillarum* or PAMPs stimulation, indicating that they might involve in the immune responses against the invaders. However, the C-type lectins exhibited agglutination towards different bacteria, for instance CfLec-2 agglutinated Gram-positive bacteria *Staphylococcus haemolyticus*, while CfLec-3 agglutinated Gram-negative bacteria *Pseudomonas stutzeri* as well as CfLec-5 agglutinated fungi *Pichia pastoris*. It was suggesting that the different C-type lectin in *C. farreri* might mediate immune response towards different pathogen through specific PAMPs recognition [22]-[25]. Furthermore, CfLec-2 could mediate cellular adhesion to enhance encapsulation of hemocytes, which was similar to CfLec-1, but it could not mediate phagocytosis [53]. All the results collectively indicated that C-type lectins in scallop were a superfamily of diverse proteins with specificity in recognition, and complementarity in function, which provided a prominent immune defense network for scallop against invaders.

## References

[1] Beutler B (2004). Innate immunity: an overview.. Mol Immunol.

[2] Loker ES, Adema CM, Zhang SM, Kepler TB (2004). Invertebrate immune systems–not homogeneous, not simple, not well understood.. Immunol Rev.

[3] Janeway CA, Medzhitov R (2002). Innate immune recognition.. Annu Rev Immunol.

[4] Medzhitov R, Janeway CA (2002). Decoding the patterns of self and nonself by the innate immune system.. Science.

[5] Christophides GK, Zdobnov E, Barillas-Mury C, Birney E, Blandin S (2002). Immunity-related genes and gene families in *Anopheles gambiae*.. Science.

[6] Yu XQ, Kanost MR (2003). *Manduca sexta* lipopolysaccharide-specific immulectin-2 protects larvae from bacterial infection.. Dev Comp Immunol.

[7] Cambi A, Figdor CG (2003). Dual function of C-type lectin-like receptors in the immune system.. Curr Opin Cell Biol.

[8] Yu XQ, Kanost MR (2000). Immulectin-2, a lipopolysaccharide specific lectin from an insect, *Manduca sexta*, is induced in response to gram-negative bacteria.. J Biol Chem.

[9] Yu XQ, Tracy ME, Ling EJ, Scholz FR, Trenczek T (2005). A novel C-type immulectin-3 from *Manduca sexta* is translocated from hemolymph into the cytoplasm of hemocytes.. Insect Biochem Molec.

[10] Koizumi N, Imamura M, Kadotani T, Yaoi K, Iwahana H (1999). The lipopolysaccharide-binding protein participating in hemocyte nodule formation in the silkworm *Bombyx mori* is a novel member of the C-type lectin superfamily with two different tandem carbohydrate-recognition domains.. Febs Lett.

[11] Tian YY, Liu Y, Zhao XF, Wang JX (2009). Characterization of a C-type lectin from the cotton bollworm, *Helicoverpa armigera*.. Dev Comp Immunol.

[12] Tunkijjanukij S, Olafsen JA (1998). Sialic acid-binding lectin with antibacterial activity from the horse mussel: Further characterization and immunolocalization.. Dev Comp Immunol.

[13] Drickamer K, Taylor ME (1993). Biology of Animal Lectins.. Annu Rev Cell Biol.

[14] Cambi A, Koopman M, Figdor CG (2005). How C-type lectins detect pathogens.. Cell Microbiol.

[15] Zelensky AN, Gready JE (2005). The C-type lectin-like domain superfamily.. Febs J.

[16] Yu XQ, Kanost MR (2001). A family of C-type lectins in *Manduca sexta*.. Adv Exp Med Biol.

[17] Liu YC, Li FH, Dong B, Wang B, Luan W (2007). Molecular cloning, characterization and expression analysis of a putative C-type lectin (Fclectin) gene in Chinese shrimp *Fenneropenaeus chinensis*.. Mol Immunol.

[18] Loukas A, Mullin NP, Tetteh KKA, Moens L, Maizels RM (1999). A novel C-type lectin secreted by a tissue-dwelling parasitic nematode.. Curr Biol.

[19] Luo T, Zhang XB, Shao ZZ, Xu X (2003). PmAV, a novel gene involved in virus resistance of shrimp *Penaeus monodon*.. Febs Lett.

[20] Bulgakov AA, Park KI, Choi KS, Lim HK, Cho M (2004). Purification and characterisation of a lectin isolated from the Manila clam *Ruditapes philippinarum* in Korea.. Fish Shellfish Immunol.

[21] Wang N, Whang I, Lee J (2008). A novel C-type lectin from abalone, Haliotis discus discus, agglutinates *Vibrio alginolyticus*.. Dev Comp Immunol.

[22] Wang H, Song LS, Li CH, Zhao JM, Zhang H (2007). Cloning and characterization of a novel C-type lectin from Zhikong scallop *Chlamys farreri*.. Mol Immunol.

[23] Zhang H, Wang H, Wang LL, Song LS, Song XY (2009). Cflec-4, a multidomain C-type lectin involved in immune defense of Zhikong scallop *Chlamys farreri*.. Dev Comp Immunol.

[24] Zhang H, Wang H, Wang LL, Song XY, Zhao JM (2009). A novel C-type lectin (Cflec-3) from *Chlamys farreri* with three carbohydrate-recognition domains.. Fish Shellfish Immunol.

[25] Zheng PL, Wang H, Zhao JM, Song LS, Qiu LM (2008). A lectin (CfLec-2) aggregating *Staphylococcus haemolyticus* from scallop *Chlamys farreri*.. Fish Shellfish Immunol.

[26] Livak KJ, Schmittgen TD (2001). Analysis of relative gene expression data using real-time quantitative PCR and the 2(T)(-Delta Delta C) method.. Methods.

[27] Smith PK, Krohn RI, Hermanson GT, Mallia AK, Gartner FH (1985). Measurement of Protein Using Bicinchoninic Acid.. Anal Biochem.

[28] Cheng SF, Zhan WB, Xing J, Sheng XZ (2006). Development and characterization of monoclonal antibody to the lymphocystis disease virus of Japanese flounder *Paralichthys olivaceus* isolated from China.. J Virol Methods.

[29] Wang DN, Liu SY, Trummer BJ, Deng C, Wang AL (2002). Carbohydrate microarrays for the recognition of cross-reactive molecular markers of microbes and host cells.. Nat Biotechnol.

[30] Yan PS, Efferth T, Chen HL, Lin J, Rodel F (2002). Use of CpG island microarrays to identify colorectal tumors with a high degree of concurrent methylation.. Methods.

[31] Yu YH, Yu YC, Huang HQ, Feng KX, Pan MM (2007). A short-form C-type lectin from amphioxus acts as a direct microbial killing protein via interaction with peptidoglycan and glucan.. J Immunol.

[32] Lin YB, Zhan WB, Li Q, Zhang ZD, Wei XM (2007). Ontogenesis of haemocytes in shrimp (*Fenneropenaeus chinensis*) studied with probes of monoclonal antibody.. Dev Comp Immunol.

[33] Ling EJ, Yu XQ (2006). Cellular encapsulation and melanization are enhanced by immulectins, pattern recognition receptors from the tobacco hornworm *Manduca sexta*.. Dev Comp Immunol.

[34] Wootton EC, Dyrynda EA, Ratcliffe NA (2003). Bivalve immunity: comparisons between the marine mussel (*Mytilus edulis*), the edible cockle (*Cerastoderma edule*) and the razor-shell (*Ensis siliqua*).. Fish Shellfish Immunol.

[35] Willment JA, Brown GD (2008). C-type lectin receptors in antifungal immunity.. Trends Microbiol.

[36] Vasta GR, Quesenberry M, Ahmed H, O'Leary N (1999). C-type lectins and galectins mediate innate and adaptive immune functions: their roles in the complement activation pathway.. Dev Comp Immunol.

[37] Watanabe A, Miyazawa S, Kitami M, Tabunoki H, Ueda K (2006). Characterization of a novel C-type lectin, *Bombyx mori* multibinding protein, from the *B. mori* hemolymph: Mechanism of wide-range microorganism recognition and role in immunity.. J Immunol.

[38] Holmskov U, Thiel S, Jensenius JC (2003). Collectins and ficolins: Humoral lectins of the innate immune defense.. Annu Rev Immunol.

[39] Torrelles JB, Azad AK, Schlesinger LS (2006). Fine discrimination in the recognition of individual species of phosphatidyl-myo-inositol mannosides from *Mycobacterium tuberculosis* by C-type lectin pattern recognition receptors.. J Immunol.

[40] Ao J, Ling E, Yu XQ (2007). Drosophila C-type lectins enhance cellular encapsulation.. Mol Immunol.

[41] Zennadi R, Chien C, Batchvarova M, Telen MJ (2006). Interaction of activated sickle red cell LW with leukocytes induces leukocyte adhesion to endothelium via CD44-E-selectin.. Blood.

[42] Frenette PS, Johnson RC, Hynes RO, Wagner DD (1995). Platelets Roll on Stimulated Endothelium in-Vivo - an Interaction Mediated by Endothelial P-Selectin.. P Natl Acad Sci USA.

[43] Lasky LA (1995). Selectin-carbohydrate interactions and the initiation of the inflammatory response.. Annu Rev Biochem.

[44] Cambi A, Gijzen K, de Vries JM, Torensma R, Joosten B (2003). The C-type lectin DC-SIGN (CD209) is an antigen-uptake receptor for *Candida albicans* on dendritic cells.. Eur J Immunol.

[45] van Vliet SJ, Gringhuis SI, Geijtenbeek TBH, van Kooyk Y (2006). Regulation of effector T cells by antigen-presenting cells via interaction of the C-type lectin MGL with CD45.. Nat Immunol.

[46] Petersen SV, Thiel S, Jensen L, Vorup-Jensen T, Koch C (2000). Control of the classical and the MBL pathway of complement activation.. Molr Immunol.

[47] Fujita T (2002). Evolution of the lectin-complement pathway and its role in innate immunity.. Nat Rev Immunol.

[48] Yu XQ, Kanost MR (2003). *Manduca sexta* lipopolysaccharide-specific immulectin-2 protects larvae from bacterial infection.. Dev Comp Immunol.

[49] Zucchetti I, Marino R, Pinto MR, Lambris JD, Du Pasquier L (2008). ciCD94-1, an ascidian multipurpose C-type lectin-like receptor expressed in *Ciona intestinalis* hemocytes and larval neural structures.. Differentiation.

[50] Weis WI, Taylor ME, Drickamer K (1998). The C-type lectin superfamily in the immune system.. Immunol Rev.

[51] Ling EJ, Yu XQ (2006). Hemocytes from the tobacco hornworm *Manduca sexta* have distinct functions in phagocytosis of foreign particles and self dead cells.. Dev Comp Immunol.

[52] Ohta M, Watanabe A, Mikami T, Nakajima Y, Kitaini M (2006). Mechanism by which *Bombyx mori* hemocytes recognize microorganisms: direct and indirect recognition systems for PAMPs.. Dev Comp Immunol.

[53] Yang JL, Qiu LM, Wei XM, Wang LL, Wang LL (2010). An ancient C-type lectin in *Chlamys farreri* (CfLec-2) that mediate pathogen recognition and cellular adhesion.. Dev Comp Immunol.

[54] Vieira OV, Botelho RJ, Grinstein S (2002). Phagosome maturation: aging gracefully.. Biochem J.

[55] Stuart LM, Ezekowitz RA (2005). Phagocytosis: elegant complexity.. Immunity.

[56] Ramet M, Manfruelli P, Pearson A, Mathey-Prevot B, Ezekowitz RA (2002). Functional genomic analysis of phagocytosis and identification of a *Drosophila* receptor for *E. coli*.. Nature.

[57] Jiravanichpaisal P, Lee BL, Soderhall K (2006). Cell-mediated immunity in arthropods: hematopoiesis, coagulation, melanization and opsonization.. Immunobiology.

[58] Beerntsen BT, James AA, Christensen BM (2000). Genetics of mosquito vector competence.. Microbiol Mol Biol R.

[59] Strand MR, Pech LL (1995). Immunological Basis for Compatibility in Parasitoid Host Relationships.. Annu Rev Entomol.

[60] Nappi AJ, Vass E (1993). Melanogenesis and the Generation of Cytotoxic Molecules during Insect Cellular Immune-Reactions.. Pigm Cell Res.

